# Recognition Code of ZNF191(243-368) and Its Interaction with DNA

**DOI:** 10.1155/2015/416751

**Published:** 2015-09-20

**Authors:** Dongxin Zhao, Zhongxian Huang

**Affiliations:** ^1^School of Chemistry and Chemical Engineering, Henan University of Technology, Zhengzhou 450001, China; ^2^Department of Chemistry, Fudan University, Shanghai 200433, China

## Abstract

ZNF191(243-368) is the C-terminal region of ZNF191 which contains a putative DNA-binding domain of four Cys_2_His_2_ zinc finger motifs. In this study, an expression vector of a fusion protein of ZNF191(243-368) with glutathione-S-transferase (GST) was constructed and transformed into *Escherichia coli* BL21. The fusion protein GST-ZNF191(243-368) was expressed using this vector to investigate the protein-DNA binding reaction through an affinity selection strategy on the basis of the binding quality of the zinc finger domain. Results showed that ZNF191(243-368) can selectively bind with sequences and react with genes which contain an AGGG core. However, the recognition mechanism of Cys_2_His_2_ zinc finger proteins to DNA warrants further investigation.

## 1. Introduction

Krüppel-type (C_2_H_2_) zinc finger is ubiquitous motif which mediates the sequence-specific recognition of DNA and widely exists in eukaryotes. This zinc finger protein can also bind DNA, RNA, or other proteins and assume critical roles in various biological functions, including cell differentiation and embryo development [1–3]. Previous studies explored the crystal/NMR structures and DNA-binding sites of zinc finger proteins, and some works reported the possibility of previewing the recognition site of a novel C_2_H_2_ zinc finger protein by sequence analysis [4–7]. However, these observations are insufficient to affirm the recognition code of each amino acid residue [8, 9]. Thus, obtaining the specific DNA-binding sequences of zinc finger proteins and understanding their functions remain challenging.

In general, the DNA-binding sequence of a novel zinc finger protein with an unknown sequence-specific DNA for recognition can be determined by random oligonucleotide selection or screening from a library of whole genomes [10–12]. However, a great deal of DNA sequences must be determined, and a consensus DNA sequence which is presumed to contact with the typical protein must be provided by computer analysis. Computation results must also be verified by experiments. The results of experiments possibly do not accord with those deduced from amino acid sequences because of the effects of several factors on the recognition code of a zinc finger protein.

The ZNF191 gene is identified from a cDNA library derived from human liver. This gene is located on human chromosome 18q12.1 and is related to a few heredity and tumor diseases [13, 14]. ZNF191 encodes a 368-amino acid protein which includes a putative DNA-binding domain of four Cys_2_His_2_ zinc finger motifs at the C-terminal region. ZNF191(243-368), the zinc finger protein of ZNF191, presumably binds to a specific DNA sequence which acts as the main functional region of ZNF191. Several studies reported on the AT-binding inclination of ZNF191(243-368). However, the actual function and DNA-binding site of ZNF191(243-368) remain unclear.

The present study aims to elucidate the function of ZNF191(243-368) at the protein level. We expressed and purified a fusion protein of glutathione-S-transferase (GST) and ZNF191(243-368) and utilized the specific affinity of GST for sepharose 4B resin to select the specific-binding DNA. Results indicated that the consensus binding site of ZNF191(243-368) had an “AGGG” core and implicated that the chemical rules for the sequence-specific recognition of Cys_2_His_2_ to DNA should be used cautiously.

## 2. Materials and Methods

### 2.1. Construction of Plasmid for Fusion Protein Expression

A cDNA fragment which encodes the zinc finger region of ZNF191 was subcloned by PCR to create a GST fusion protein. The following oligonucleotide primers (Union Genetic Company, Shanghai, China) were used for PCR and cloning: P1 (5′-CGCGGATCCAGAAATCCCTCTCGAAAGAAACAA-3′); P2 (5′-TCCCCCGGGTTAAACTTCCACAACATTCAGAAG-3′). The PCR template was pTSA-ZF vector [14]. The ZNF191(243-368) gene was amplified using the upper primer P1 (introduced Bam HI site) and the lower primer P2 (introduced Sma I site). The PCR-amplified fragment and pGEX-4T-2 (Amersham Pharmacia) vector were incubated with Bam HI and Sma I, respectively, purified by an electrophoresis gel, and then mixed and ligated using T4 ligase (BioLabs). The ligation product was transformed into BL21 host strains. The bacterial clone was sequenced, and the recombinant vector was named pGEX-ZF [15].

### 2.2. Overproduction and Purification of Zinc Finger Fusion Protein

LB medium (50 mL) containing 100 *μ*g/mL ampicillin was inoculated with a single freshly picked colony which contains the expression plasmid pGEX-ZF and then incubated overnight at 37°C. The overnight culture was diluted 100 times in 500 mL of 2YT medium and then grown at 37°C to OD_600_ = 0.6. Isopropylthio-*β*-D-galactoside was then added to a final concentration of 0.5 mM, and cells were induced for 3 h at 30°C. The cells were harvested by centrifugation (6,000 rpm and 4°C), resuspended in 100 mL ice cold PBS (pH 7.4) with 10 mM *β*-mercaptoethanol, and then lysed by lysozyme at 4°C for approximately 30 min. The mixture was added with 1% Triton X-100, 1 mM PMSF, and 5 U/mL DNase I (Sango Company, Shanghai, China) with stirring for 30 min at 4°C to aid protein solubilisation and then centrifuged at 15000 rpm for 30 min at 4°C. The supernatant was mixed with 10 mL of glutathione sepharose 4B slurry (Amersham Pharmacia Biotech) and then shaken for 30 min. The affinity matrix was extensively washed with PBS until OD_280_ < 0.02, and then GST fusion proteins were eluted in an elution buffer (50 mM Tris, 100 mM reduced glutathione, pH 8.0). The eluted solution was concentrated using Amicon YM-10 (Millipore) and passed through Sephadex G75 column. The purified fusion proteins were mixed with resin for the following binding experiment.

### 2.3. Zinc Finger Protein-DNA-Binding Assay

Bound DNA of ZNF191(243-368) was obtained using a pool of random oligonucleotides. A 60-base single-strand DNA oligonucleotide which contains a central region of 18 random bases flanked by a 21-base region with defined sequences was synthesized as a template with a sequence of 5′-ATTCAGATCTTAAACACAGGA…N^18^…GTGATGCTCGGTACCCTAAAG-3′. The following primers were used for PCR: A1 (5′-CGCGGATCCATTCAGATCTTAAACA-3′) and A2 (5′-TTCCCCGGGCTTTAGGGTACCG-3′). A mixture of double-stranded DNA fragments was obtained by Pfu polymerase (BioLabs) through 10 cycles of denaturation (94°C, 1 min), annealing (56°C, 1 min), and extension (72°C, 1 min). The PCR products were initially treated with phenol/CHCl_3_ and then precipitated with ethanol. These DNAs were incubated with GST-ZNF191(243-368) fusion proteins bound to glutathione sepharose 4B (prepared as previously described) at 4°C for 30 min in binding buffer [0.2 mg/mL poly(dI-dC) (Sigma), 0.2 mg/mL BSA (BioLabs), 25 mM HEPES (pH 7.5), 100 mM KCl, 0.1 mM ZnSO_4_, 10 mM MgCl_2_, 0.1% Nonidet P-40, 1 mM DTT, and 5% glycerol] [11]. The resin beads were centrifuged and washed four times with binding buffer. The bound oligonucleotides were eluted from beads using the elution buffer and then boiled for 10 min in H_2_O. After centrifugation, the supernatant was used for PCR amplification using primers P1 and P2. After three rounds of selection amplification, the PCR products were digested with Bam HI and Sma I and cloned into pGEX-4T-2 vectors. The recombinant vectors were transformed into* Escherichia coli*, and the obtained colonies were sequenced.

### 2.4. UV-Vis Absorption Spectroscopy

The UV spectra of GST-ZNF191(243-368) fusion protein, GST, and ZNF191(243-368) were recorded on an HP 8453 Diode Array spectrophotometer (USA). The concentration of the protein solution (pH 7.5) with 10 mM Tris-HCl was detected via Bradford's method, which was 1 *μ*mol·L^−1^.

### 2.5. Circular Dichroism (CD) Spectroscopy

Circular dichroism spectra of GST-ZNF191(243-368) fusion protein, GST, and ZNF191(243-368) were recorded between 190 and 250 nm on a J720 Jasco spectropolarimeter. The optical path length was 10 mm, and the concentration of the protein solution (pH 7.5) with 10 mM Tris-HCl was 1 *μ*mol·L^−1^. The recordings were conducted at 25°C.

### 2.6. Fluorescence Measurements

Two synthesized and purified DNA duplexes were used to probe for the DNA-binding activity of ZNF191(243-368) by using fluorescence spectroscopy. One DNA contains the obtained sequence GGAGGGTGGTTA (DNA1), and the other contains the 12 bp motif GAAATAATGTTA (DNA2), as predicted by previous reports [8].

DNA-binding studies were performed in a buffer (pH 8.0) which contains 50 mM Tris-HCl and 10 mM NaCl. Titration processes were conducted by adding protein stock solution, GST-ZNF191(243-368), or GST to 1 mL of a buffer which contains 500 nmol·L^−1^ of the 12 bp oligonucleotide duplex and 1 *μ*mol·L^−1^ ethidium bromide (EB).

Fluorescence emission spectra were obtained on a Cary Eclipse fluorescence spectrometer (Varian Company) within 550–700 nm at an excitation wavelength of 540 nm in a 1 cm × 0.5 cm fluorescence cuvette at 20°C. The entrance and exit slits for all fluorescence measurements were maintained at 10 nm.

## 3. Results and Discussion

### 3.1. Construction of Plasmid and Expression of Fusion Protein

**Figure 1:**
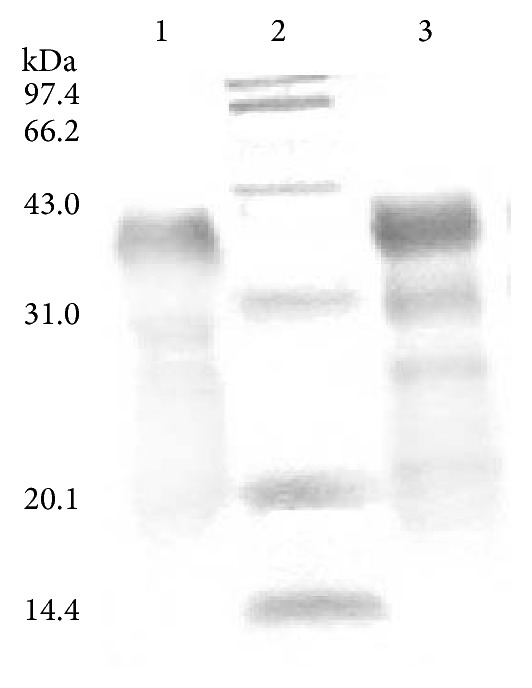
SDS-PAGE of GST-ZNF191(243-368) fusion protein. Lane 1: protein purified by G75, lane 2: protein marker, and lane 3: protein purified by affinity resin.

From the DNA sequence of the recombinant plasmid pGEX-ZF, the N-terminus of the ZNF191(243-368) gene was linked with the C-terminus of the GST gene. Thus, this fusion gene expressed a 40 kDa protein which contains four C_2_H_2_-type tandem zinc finger motifs at the C-terminus of the fusion protein. Bacterially expressed GST-ZNF191(243-368) was purified by glutathione sepharose 4B and Sephadex G75 column in accordance with a previously described method. SDS-PAGE analysis of the purified proteins shows a prominent band of the expected size for fusion proteins (Figure 1). After the purified fusion protein was bound to the resin, the mixture was used for the following binding experiment.

### 3.2. Affinity-Based Selection of DNA-Binding Sites

**Figure 2:**
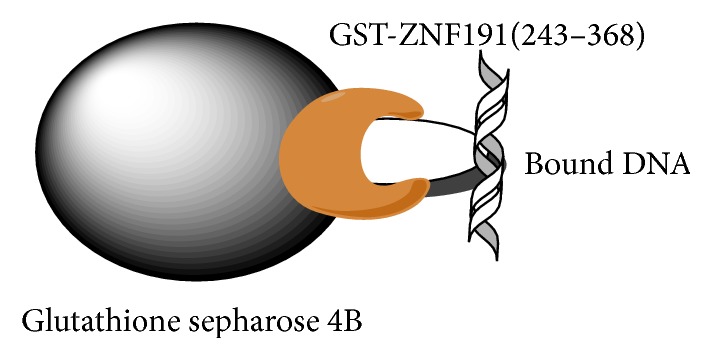
Schematic representation of DNA selecting of GST-ZNF191(243-368). Glutathione sepharose 4B is bound by the GST of fusion protein. Meanwhile, the zinc finger protein binds its recognition sequence in DNA and thus immobilizes the DNA on glutathione sepharose 4B.

The predicted structures of four C_2_H_2_ zinc finger domains and gene localisation suggest that ZNF191(243-368) is a DNA-binding protein. To determine the DNA-binding site of ZNF191(243-368), we used the random oligonucleotide binding selection strategy as previously described. Two affinity processes are involved in the selection procedure, namely, the binding of GST-ZNF191(243-368) to matrix immobilised glutathione sepharose 4B and the binding of the random oligonucleotides to the zinc finger protein (Figure 2). A similar binding experiment of the random oligonucleotides to the GST protein was performed as a control.

**Figure 3:**
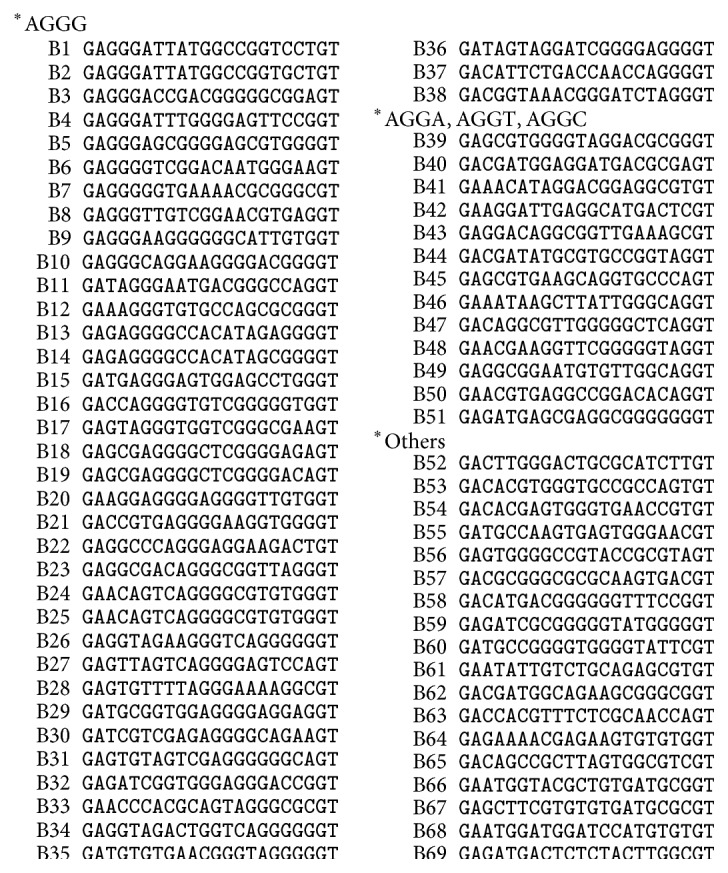
Alignment of 69 selected oligonucleotides bound to ZNF191(243-368). There are 51 sequences (74%) containing AGGG motif or similar AGGA, AGGT, AGGC motif, and the content of GC in others is high.

Based on affinity, we obtained the DNA-binding site of ZNF191(243-368) through a series of washing and amplification processes. We finally transferred these DNA sequences into the pGEX vector and then sequenced this plasmid. DNA sequences which strongly bind to the protein were obtained (Figure 3).

### 3.3. DNA-Binding Activity

The sequences of the selected DNA-binding sites for GST-ZNF191(243-368) were compared. Among the 69 clones, 38 (55%) had an AGGG sequence, and similar sequences AGGC, AGGT, and AGGA were present in 7, 4, and 2 clones, respectively. Thus, 51 (74%) of the resulting clones were discovered to contain an “AGG” core. Although the “AGG” core is outside residual sequences, several sequences have the similar sequences AGCG and ACGG. In addition, the content of GC is high. These results suggest that GST-ZF binds more strongly to the AGGG sequence than to the other sequences.

After comparing these sequences which contain AGGG and computing the probability of bases before and after AGGG, we regarded the binding sequence to be (G)(G)AGGG(G) (Table 1).

The bases of binding sequence were designated 1 through 8 in the binding site column. Comparing these sequences containing AGGG which sometimes appeared two times in a sequence, the number in the bases selected column indicates the frequency of each base that appeared at the 1–8 positions. Each number in the percentage columns indicates the percentage of selected oligonucleotides containing that base at the indicated position.

### 3.4. Eukaryotic Promoter Database

ZNF191 is widely expressed in human organs and is possibly related to a few hereditary and cancer diseases. Thus, the genes which contain the AGGGG core are possibly the target genes of ZNF191(243-368). We searched the genes that contain GGAGGGG in the Eukaryotic Promoter Database [16]. Many gene promoters contain this binding site of ZNF191(243-368), such as AHSA1, NOSIP, RHO, and HIVEP2 which relate to metastatic prostate cancer, neuronal NOS activity, denatured states of rhodopsin, and immunodeficiency, respectively [17–20]. Otherwise many chromosome open reading frames, docking proteins, transcription factors, and zinc finger proteins also contain this sequence [21–24]. These indicated ZNF191 widely expressed in human organs possibly related to many physiological processes and diseases.

The genes which contain ZF-binding sites possibly react with ZNF191(243-368). Nevertheless, we cannot exclude another possibility that the DNA sequence is not the biological binding site of this protein. Apart from the zinc finger domain of a protein which affects the DNA-binding properties, a few residues outside the zinc finger domain are also involved in the recognition. In addition, the known chemical rules based on the zinc finger motif should be used cautiously. Further studies on the ZNF191 zinc finger protein with DNA are underway.

### 3.5. UV-Vis Absorption Spectroscopy

**Figure 4:**
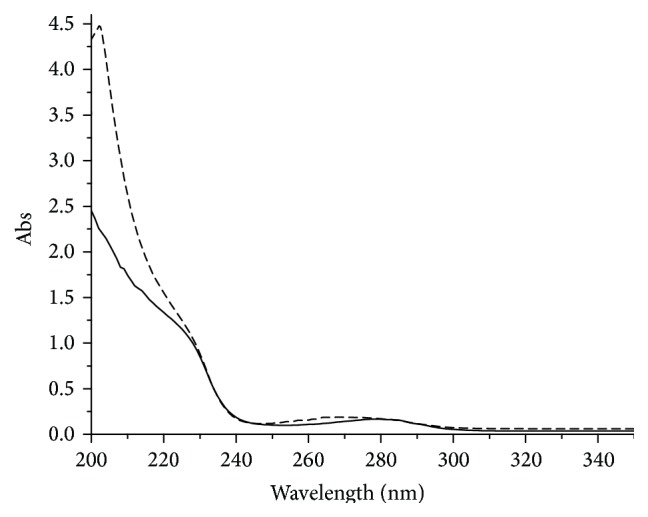
Different UV spectrum of GST-ZNF191(243-368) and GST. Solid line is UV spectrum of ZNF191(243-368) and dash line is different UV spectrum of GST-ZNF191(243-368) and GST.

The different UV spectrum of the GST-ZNF191(243-368) fusion protein and GST were obtained by subtracting the UV spectrum of GST from that of GST-ZNF191(243-368) (Figure 4). The result indicates that the ZNF191(243-368) in the fusion protein maintains its structure for the absorption of aromatic amino acid residues at 260 nm to 280 nm and the absorption of Zn-S bond at approximately 230 nm [25, 26]. Furthermore, the absorption of several chromophores in GST beyond the linear range cannot be fully deduced from the spectrum, which leads to the significant difference at 200 nm.

### 3.6. CD Spectral Measurements

**Figure 5:**
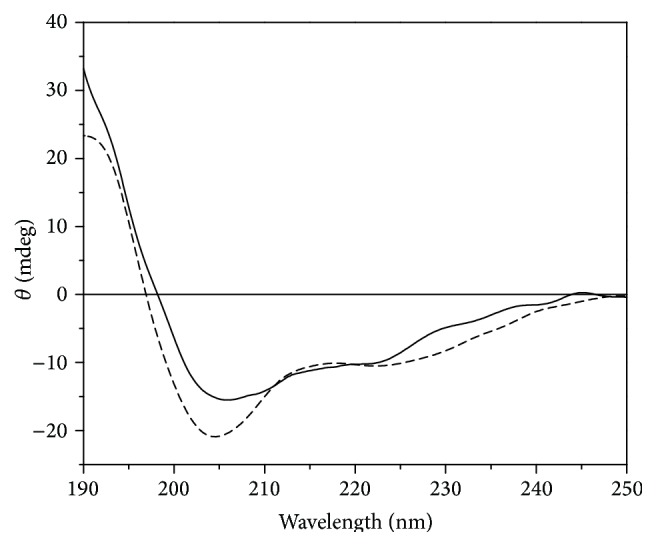
Different CD spectrum of GST-ZNF191(243-368) and GST. Solid line is CD spectrum of ZNF191(243-368) and dash line is different CD spectrum of GST-ZNF191(243-368) and GST.

The different CD spectrum of GST-ZNF191(243-368) and GST shows that the ZNF191(243-368) in the fusion protein also has two negative peaks at approximately 210 and 220 nm and a positive peak at approximately 190 nm of the *α*-helix (Figure 5). Therefore, the ZNF191(243-368) in the fusion protein also maintains a second structure [27–30].

### 3.7. Binding Constant of Protein-DNA Complexes

Fluorescence spectroscopy is widely used to probe protein-DNA interactions. The binding curves can be generated by titrating the protein solution into DNA solution, and the dissociation constant (*K*_*d*_) can be obtained by analysing the resulting curve. The addition of EB into the DNA solution enhances the changes in fluorescence intensity.

EB and ZNF191(243-368) compete to bind with DNA in the three-component system on the basis of the Scatchard Equation: *r*/*c* = *K*(*n* − *r*) [31–33]. The associations can be described as

(1) rEcE=n−rEKE1+KPcP,

(2) rPcP=n−rPKP1+KEcE,

where *r*_*E*_ and *r*_*P*_ are the ratios of bound EB or protein concentration to the total concentration of DNA; *c*_*E*_ and *c*_*P*_ are the concentrations of free EB and protein, respectively; *n* is the binding number in each oligonucleotide; and *K*_*E*_ and *K*_*P*_ are the association constants of EB and protein with DNA, respectively.

From (1) and (2), (3) can be obtained as follows:

(3) $$\begin{array}{c} \left(\;n-r_{E}\;\right)K_{E}\left(\;\frac{c_{E}}{r_{E}}\;\right)-1=r_{P}\left[\;\frac{\left(1+K_{E}c_{E}\right)}{\left(n-r_{P}\right)}\;\right] \end{array}$$

and *c*_*E*_ = [(*F*_0_ − *F*)/(*F*_0_ − *F*_*∞*_)]*c*_*E*0_, in which *F*_0_ and *F* are the fluorescence intensities of the EB-DNA system without and with protein, respectively, and *c*_*E*0_ is the total concentration of EB, which is a known quantity. Thus, only *r*_*P*_ is the unknown in (3). Based on the formula *c*_*P*_ = *c*_*P*0_ − *r*_*P*_ *c*_*D*0_, we can calculate and substitute *r*_*P*_ into (2) to obtain the binding constant *K*_*P*_ of protein and DNA.

**Figure 6:**
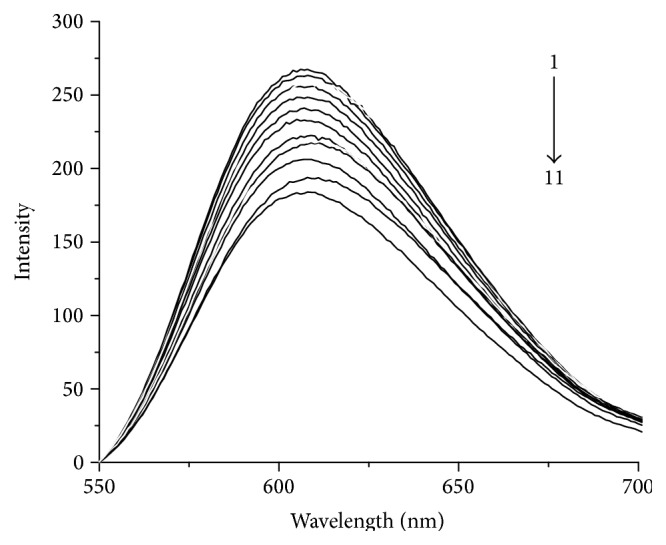
Fluorescence spectra of EB-DNA1 titrated by ZNF191(243-368). *C*_DNA_ = 0.5 *μ*mol·L^−1^, *C*_EB_ = 1 *μ*mol·L^−1^, from up to down, and *C*_protein_/*C*_DNA_ = 0, 0.1, 0.2, 0.3, 0.4, 0.5, 0.6, 0.7, 0.8, 0.9, 1.0, respectively.

Figure 6 shows the fluorescence spectra of the fusion protein and DNA. In this system,* n* and *K*_*E*_ are 0.2 and 5.0 × 10^7^ L·mol^−1^, respectively [34, 35]. Thus, the association constant *K*_*P*_ of GST-ZNF191(243-368) with DNA can be calculated by the equations. The results are shown in Table 2.

The zinc finger protein more strongly binds to DNA1 than to DNA2. In addition, the binding result of DNA2 is close to previous studies, in which two mutant proteins of ZNF191(243-368) were purified from the pTSA/BL21(DE3) system [15]. Thus, the fusion protein can be used to study protein-DNA interactions by the fluorescence method of the three-component system with the highly sensitive and selective EB fluorescence probe. Furthermore, the binding constant between the zinc finger protein and DNA1 obtained from this experiment is 3.8 × 10^7^ L·mol^−1^, which is of nearly one order of magnitude higher than that of the zinc finger protein and DNA2, and very close to those of other zinc finger proteins and DNA [36, 37]. Therefore, we deduced a strong interaction between ZNF191(243-368) and GGAGGG, which can provide useful information for understanding the function of ZNF191(243-368).

However, this result is different from that reported by Yu et al. This difference may be attributed to the effects of several factors on the recognition of zinc finger protein to DNA. For example, critical residue(s) in the rest of ZNF191 can induce DNA bending, which facilitates the easy binding of the zinc finger protein [38–40]. The SCAN box located before ZNF191(243-368) also has important functions in DNA recognition [41, 42]. Moreover, the cooperative interaction of the various protein components is a factor [43, 44].

## 4. Conclusion

ZNF191(243-368) can selectively bind with sequences and react with genes which contain an AGGG core. But the zinc finger domain of a protein is not the only factor which affects DNA-binding properties; some residues outside the zinc finger domain are also involved in the recognition. The known chemical rules based on the sequence of the zinc finger motif should be used cautiously. Further studies on the ZNF191 zinc finger protein with DNA are underway.

## Figures and Tables

**Table 1:** Derivation of the ZNF191(243-368) consensus binding site.

Base selected				Percentage (%)				Binding site
G	C	A	T	G	C	A	T	5′-3′
20	7	8	9	45	16	18	20	G
24	9	4	7	55	20	9	16	G
0	0	44	0	0	0	100	0	A
44	0	0	0	100	0	0	0	G
44	0	0	0	100	0	0	0	G
44	0	0	0	100	0	0	0	G
23	4	11	6	52	9	25	14	G
14	11	9	10	32	25	20	23	G/C/A/T

**Table 2:** The value of *K*_*P*_ of ZNF191 (243-368) and DNA.

	*K*_*P*_ (L/mol)
GST-ZNF191(243-368)/DNA1	3. 8 × 10^7^
GST-ZNF191(243-368)/DNA2	5.4 × 10^6^
I323W^a^	3.2 × 10^6^
R327W^a^	3.6 × 10^6^

^a^Obtained from pTSA/BL21(DE3) system; it is the mutants of ZNF191(243-368), and used DNA sequence is similar to DNA2.
